# The Alumni Association of the Medical Department of the University of Buffalo

**Published:** 1874-12

**Authors:** 


### Editorial.

### The Alumni Association of the Medical Department of the University of Buffalo.

The arrangements for the Meeting of the Alumni and Officers of the Medical Department of the University of Buffalo, which is to be held in February next, in connection with the Annual Commencement of the College, are being made with zeal and care. We are informed by the committee who have the matter in charge, that they have so far perfected their arrangements and plans, that they feel assured of having a successful meeting both in a business and scientific as well as social way. Besides the transaction of business which it is proposed shall take place in the afternoon of the day of Commencement, there is to be delivered a public address to the Alumni in the evening in St. James Hall, delivered in connection with the Graduation exercises, succeeding the presentation of Diplomas and the Address by the Faculty to the graduates of the present session. The orator for the Alumni has accepted his appointment, and it may be expected that the exercises of the occasion will be interesting and largely attended by physicians and by the public. Arrangements for a social gathering and entertainment following the public exercises have been instituted.

Invitations are as far as possible sent the members of the Alumni by the Committee, but owing to the imperfections in the list of addresses which they now have, many may fail to receive personal invitation from them, and the committee desire us to give in the JOURNAL a general invitation to all interested to make an endeavor to be present and contribute their part to the interest and pleasure of the meeting. The programme in particular will be given in our next number.

